# Blue Light-Activated Riboflavin Phosphate Promotes Collagen Crosslinking to Modify the Properties of Connective Tissues

**DOI:** 10.3390/ma14195788

**Published:** 2021-10-03

**Authors:** Yeyoung Kang, Jae Hak Kim, Seo Young Kim, Won-Gun Koh, Hyun Jong Lee

**Affiliations:** 1 Department of Chemical and Biological Engineering, Gachon University, 1342 Seongnam-daero, Seongnam-si 13120, Korea; yeyounglab@gmail.com (Y.K.); jaehak96@gmail.com (J.H.K.); seoyk20@gmail.com (S.Y.K.); 2 Department of Chemical and Biomolecular Engineering, Yonsei University, 50 Yonsei-ro, Seodaemun-gu, Seoul 03722, Korea

**Keywords:** collagen, photo-crosslinking, skin elasticity, riboflavin phosphate, hydrogel

## Abstract

Reduced amounts of collagen and fragmented collagen fibers are characteristics of aging skin. Recently, user-friendly, at-home personal aesthetic devices using light-emitting diode (LED) light have been used for cost-effective and safe skin improvement. However, to dramatically improve the skin via collagen repair, we need to develop an LED-responsive photosensitizer. Corneal collagen crosslinking uses ultraviolet light to activate riboflavin phosphate (RFP) and is used in ophthalmology. RFP is a biocompatible photosensitizer derived from vitamin B_2_. This study aimed to prove that RFP combined with blue light (BL) can increase collagen crosslinking density, improving its mechanical properties in skin tissue and enhancing skin elasticity. We confirmed the RFP-induced photo-crosslinking in pure collagen by studying changes in its dynamic modulus and matrix morphology using collagen hydrogels. We also measured the changes in the mechanical properties after applying photo-crosslinking on porcine skin. The Young’s modulus (1.07 ± 0.12 MPa) and tensile strength (11.04 ± 1.06 MPa) of the porcine skin after photo-crosslinking were 2.8 and 3.5 times better compared to those of normal porcine skin, respectively. Thus, photo-crosslinking through RFP and BL irradiation can be potentially used for skin improvement using aesthetic LED devices.

## 1. Introduction

As we age, our skin changes too. Skin aging manifests as wrinkles, pigmentation, telangiectasia, and loss of elasticity. At the molecular level, aging skin tissues exhibit decreased collagen and fragmented collagen fibers [1]. Several methods have been used to promote collagen biosynthesis and remodel the dermal layer to restore the aging collagen matrix, including applying retinoic acid, dermabrasion, chemical peeling, and ablative laser therapy [2,3,4,5]. However, these methods require intensive post-treatment care and prolonged downtime because of the possibility of complications such as erythema, pain, infection, burns, pigmentation, and scarring [6,7,8]. Therefore, we need to develop safer and more effective alternative rejuvenation procedures that overcome the limitations of the existing methods.

As personal aesthetic devices using light-emitting diodes (LEDs) gained popularity, their use for skin rejuvenation also increased. LED, a nonthermal nonablative light source, is widely used in at-home cosmetic/medical devices as it is cheaper, safer, and easier to use than the existing laser sources [9,10,11]. Many studies have been conducted in vitro and in vivo on the biological response to different LED wavelengths, intensities, and irradiation time [8,9,10]. The research has shown that LED light therapy can improve the quality of the skin by cell activation. However, a new approach using LED light is needed to enhance collagen repair dramatically, such as LED-responsive photosensitizer.

Riboflavin phosphate (RFP) is a water-soluble form of riboflavin (vitamin B_2_). It has been studied extensively as a photosensitizer. In 2016, U.S. Food and Drug Administration (FDA) approved the use of RFP with ultraviolet (UV) light for collagen crosslinking in ophthalmology [12,13,14]. In the procedure, after the RFP solution is applied and absorbed into the tissues, the skin is irradiated with weak UV light for 30 min to increase the crosslinking density of the collagen matrix in the corneal tissue. It is the only proven photosensitizer-based clinical treatment for protein crosslinking. We can also apply RFP and UV radiation to skin collagen to increase skin elasticity. However, UV rays can harm the skin; therefore, we need an alternative light source. Previous studies have confirmed that blue light (BL) can activate RFP and can be used as an alternative to UV light [15]. In addition, BL can penetrate deeper into the skin tissues. Therefore, theoretically, LEDs used with BL-activated RFP will have a greater impact on collagen repair and skin improvement compared to LEDs used alone.

This study aimed to investigate the possibility of using RFP-induced collagen crosslinking to improve skin elasticity and determine the optimal reaction conditions. We replaced UV with BL, which is a safer RFP activation light source in the collagen crosslinking. To quantify the improvement in elasticity, we measured the mechanical properties of porcine skin tissue after treating it with RFP solution and irradiating it with BL.

## 2. Materials and Methods

### 2.1. Materials

Unless otherwise mentioned, all chemicals and solvents were used as instructed by the manufacturer. Collagen I, bovine (5 mg/mL), and phosphate-buffered saline (PBS, pH 7.4) were purchased from Gibco (Waltham, MA, USA). Sodium hydroxide solution (NaOH, 1.0 N), RFP, and pH test strips (pH 4.5–10) were purchased from Sigma Aldrich (St. Louis, MO, USA); 10 X PBS (pH 7.4) was purchased from Invitrogen (Carlsbad, CA, USA) and, the Micropig^®^ Franz cell membrane (5 cm × 5 cm × 400 μm) was purchased from Apures (Gyeonggi, Korea).

### 2.2. Fabrication of Crosslinked Collagen Hydrogels

We mixed DI water (1425 µL), 10 X PBS (500 µL), and NaOH (75 µL) to create the neutralization solution. Collagen I was added to the neutralization solution in a 3:2 volume ratio. The RFP solution was prepared by dissolving RFP in PBS. It was then mixed with the neutralized collagen solution to get the final concentration of 0.01 wt%. For the gelation process, we irradiated the RFP-added, neutralized collagen solution with BL (commercial 1.5 W/cm^2^ dental LED curing light) for 1, 5, 10, 20, and 30 min. We also synthesized a physically crosslinked collagen hydrogel without RFP. The crosslinked collagen hydrogels were incubated at 37 °C for 3 h to complete the gelation.

### 2.3. Fourier Transform Infrared (FT-IR) Spectroscopy

The characteristic chemical structure change of collagen hydrogels by chemical crosslinking was measured with the Fourier transform-infrared (FT-IR) spectroscopy (Thermo Fisher Scientific iS50, Waltham, MA, USA). All spectra were recorded within the wavelength range of 600–1800 cm^−1^. For comparison, the physically crosslinked collagen hydrogel and BL-activated RFP-induced chemically crosslinked collagen hydrogels were freeze-dried and analyzed.

### 2.4. Rheological and Morphological Characterization of Crosslinked Collagen Hydrogels

We used rheological measurements to determine the RFP concentration required to produce crosslinked hydrogels with improved mechanical properties (MCR 301, Anton Paar, Germany). We prepared the collagen hydrogels of 10-mm diameter, mounted them onto the rheometer. First, the storage and loss moduli were measured using amplitude sweeps from 0.1% to 10% with a fixed 1 Hz frequency to determine the linear viscoelastic region. Then, the storage and loss moduli were measured using frequency sweeps from 0.1 to 10 Hz with a fixed 1% strain. The temperature was maintained at 37 °C during the experiment.

The structure and morphology of the collagen hydrogels were observed using scanning electron microscopy (SEM; Hitachi S-4700, Tokyo, Japan). We prepared the cross-sections of the collagen hydrogels by freezing them in liquid nitrogen and breaking the frozen hydrogel samples into segments and lyophilizing them. The analytes were coated with platinum under vacuum before SEM measurements.

### 2.5. Characterization of the Mechanical Properties of Crosslinked Collagen in Porcine Skin

The Micropig^®^ Franz cell membranes were cut into rectangles (5 cm × 1 cm) and incubated in RFP solutions of concentrations 0.01, 0.05, and 0.1 wt% for 1 h. After removing them from RFP solutions, the skin surfaces were gently washed with PBS and irradiated with BL for 1, 5, 10, and 30 min. We measured the mechanical properties using a Universal Testing Machine (UTM 34sc-1, Instron, Canton, MA, USA) at the Smart Materials Research Center for IoT, Gachon University. The strain rate was set to 1 cm/min using a 5 N load cell until breakage (*n* = 3). The Young’s modulus and tensile strength of each sample were measured using the software accompanying the Instron.

### 2.6. Statistical Analysis

All data are expressed as mean ± standard deviation (SD). Each experiment was repeated three times unless otherwise indicated. GraphPad Prism was used to plot the trend line of crosslinked collagen hydrogel storage modulus versus BL irradiation time by simple linear regression. In addition, the trend lines of Young’s modulus and tensile strength of porcine skins versus irradiation time were plotted by Sigmoidal four-parameter logistic (4PL).

## 3. Results and Discussion

### 3.1. Collagen Crosslinking Promoted by BL-Activated RFP

**Figure 1:**
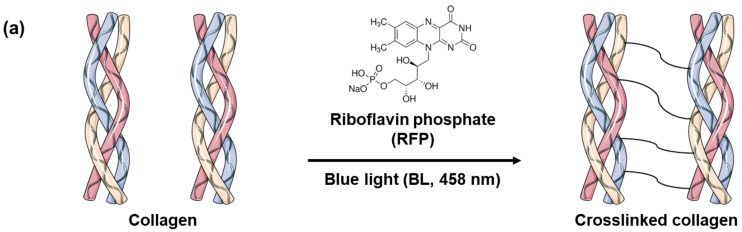

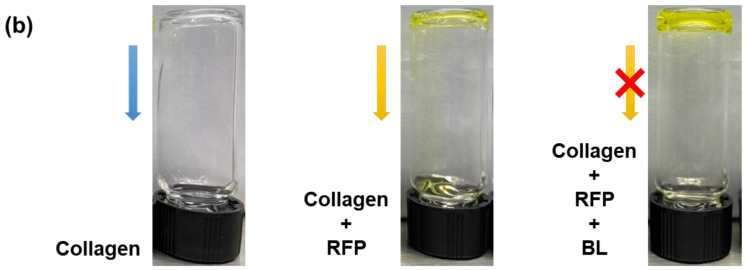
(**a**) Schematics of collagen crosslinking through blue light (BL)-activated riboflavin phosphate (RFP). (**b**) Photo images of collagen solution without RFP and collagen solutions with RFP before and after BL irradiation.

Skin elasticity can be improved by increasing the collagen crosslinking density in the skin tissues. However, before using BL-activated RFP to induce collagen crosslinking in the skin, it is necessary to understand its effects on pure collagen. Therefore, we investigated the physical and chemical changes in pure collagen upon exposure to BL-activated RFP. We quantified collagen crosslinking by observing the changes in the mechanical properties of the collagen hydrogel (Figure 1). Initially, the neutralized collagen solution without RFP was transparent with high viscosity but still flowing. After adding RFP to the collagen solution, it turned yellow but showed no difference in viscosity. However, after BL irradiation, the collagen formed a white, turbid hydrogel, which remained fixed at the bottom of the vial without flowing, confirming the crosslinking of collagen by BL-activated RFP.

RFP, a derivative of vitamin B_2,_ is a non-toxic compound that has been used in light-induced protein crosslinking in clinical treatments [13,14]. The process for collagen crosslinking is simple. The solubilized RFP is applied to the tissues, which are then irradiated for a few minutes to activate the photosensitizer. UV and BL can activate RFP, as RFP absorbs both UVA and BL with maxima at 380 and 450 nm, respectively. Collagen contains several amino acids with reactive side chains, such as tyrosine, tryptophan, histidine, lysine, methionine, and cysteine resulting in multiple reaction pathways for photosensitized crosslinking [16,17]. The activated RFP generates singlet oxygen (^1^ O_2_), which oxidizes the reactive side chains of the amino acids. The activated side chain then crosslinks with another amino acid side chain [18,19]. Alternatively, the carbonyl and amine groups of the amino acids can covalently bond after singlet oxygen activation [20]. The photoreaction of RFP links the collagen fibrils with the dominant reaction pathway depending on the type of collagen and the reaction conditions [21].

**Figure 2:**
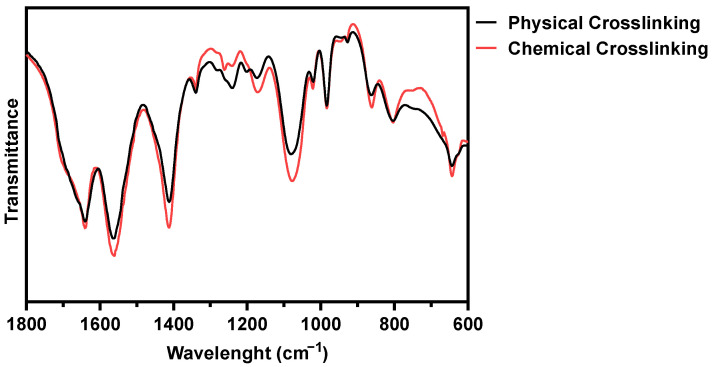
FT-IR spectra of physically and BL-activated RFP-induced chemically crosslinked collagen hydrogels.

The impact of BL-activated RFP on collagen construct was evaluated by Fourier transform infrared (FT-IR) spectroscopy (Figure 2). The physical and chemical crosslinked hydrogel showed amides I and II absorptions could be found at 1640 and 1560 cm^−1^, respectively. After chemical crosslinking, the intensity of the peaks was slightly enhanced, which was attributed to C = N stretching [22]. The peak at 1240 cm^−1^ represents the C-N stretching of amine, and the peak was weakened after chemical cross-linking. The amine group was consumed for the formation of covalent bonds. The chemical crosslink increased the peak at 1170 cm^−1^, representing the C-O stretch of the ester group. The singlet oxygen with high energy induces covalent bonds between proline residues and histidine residues in adjacent chains, and the bonds form an additional C-O ester group [23]. The possibility of the progress of the two reactions, carbonyl-amine and proline-histidine, was confirmed through FT-IR.

### 3.2. Rheological Characterization of Crosslinked Collagen Hydrogels

**Figure 3:**
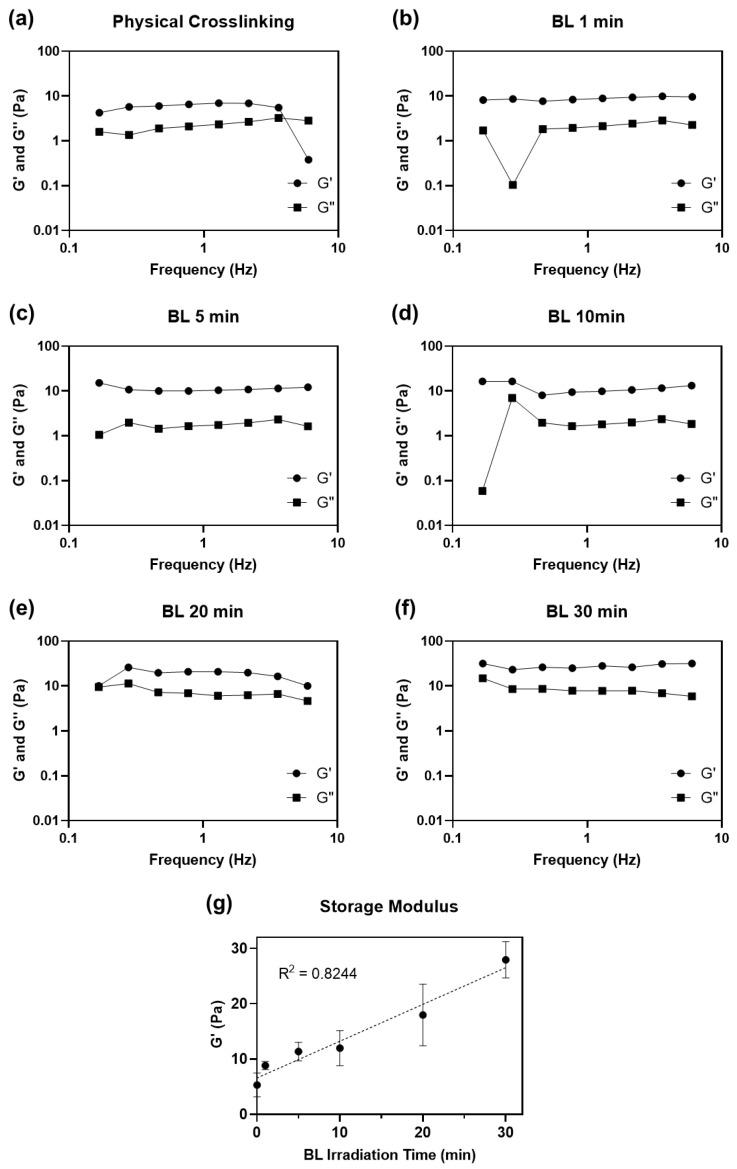
Dynamic moduli (G′: storage modulus and G″: loss modulus) plotted as a function of the frequency of (**a**) physically and (**b**–**f**) BL-activated RFP-induced chemically crosslinked collagen hydrogel from 0.1 to 10 Hz of frequency. (**g**) Average storage moduli of crosslinked collagen hydrogel.

We compared the mechanical properties of physically and chemically crosslinked collagen by measuring the dynamic moduli of the hydrogels (Figure 3).

We also evaluated the effect of irradiation time (1 to 30 min) on the mechanical properties of the hydrogels. All collagen hydrogels were incubated at 37 °C for 3 h to ensure complete gelation after neutralization and irradiation steps before measuring their dynamic moduli. All collagen matrices, including physically crosslinked collagen hydrogel without BL-activated RFP, had higher storage moduli (G′) than loss moduli (G″), indicating that all collagen solutions formed hydrogels (Table 1). The hydrogel crosslinked with activated RFP and irradiated for 1 min showed a higher storage modulus (8.82 ± 0.72 Pa) than that of the physically crosslinked hydrogel (5.28 ± 2.03 Pa) irradiated for the same time. Although the 1 min irradiation time was short, the storage modulus of the hydrogels increased by 1.7 times compared to that of physically crosslinked collagen. The storage moduli increased with BL irradiation time. After 30 min of BL irradiation, the storage modulus of the collagen hydrogel was 27.92 ± 3.09, approximately 5.3 times higher than that of the physically crosslinked hydrogel.

The neutralized collagen solution forms a physically crosslinked hydrogel through spontaneous self-assembly of collagen molecules around 37 °C [24]. However, physically crosslinked collagen has weak mechanical properties. Intrahelical and intermolecular covalent bonds, formed between collagen molecules using photosensitized chemical crosslinking, increased the mechanical strength of collagen. Longer light irradiation time creates more singlet oxygen molecules, leading to denser crosslinking. Barring few outliers, the storage modulus showed a linear relationship with BL irradiation time (R^2^ = 0.8244). The storage modulus did not saturate even after irradiating the hydrogel for 30 min.

### 3.3. Morphological Characterization of Crosslinked Collagen Hydrogels

**Figure 4:**
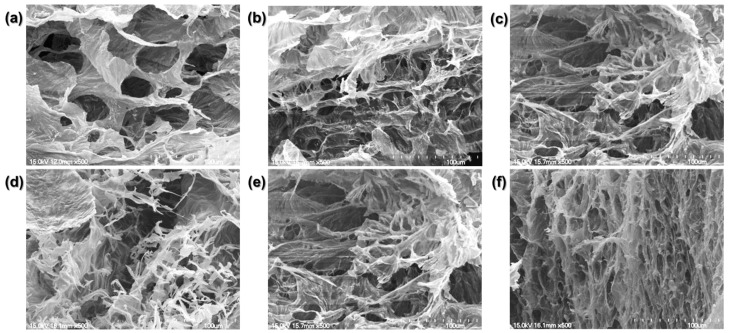
Scanning electron microscopy (SEM) images. (**a**) Physically crosslinked hydrogel. (**b**) 1 min, (**c**) 5 min, (**d**) 10 min, (**e**) 20 min, and (**f**) 30 min BL-activated RFP-induced chemically crosslinked collagen hydrogels.

We compared the crosslinked hydrogel structures using the SEM images (Figure 4). 

The physically crosslinked collagen matrix is porous with flat planes connecting the pores. The chemically crosslinked collagen hydrogel was mesh-like with lesser flat planes. As the BL irradiation time increased, the planar structure decreased, and the mesh density increased. Consistent with the similar dynamic moduli of the hydrogels irradiated for 1, 5, 10, and 20 min, the four groups showed similar matrix structures. Their structures consisted of broad flat planes with a mesh-like arrangement of twisted fibrils. However, the hydrogel irradiated for 30 min exhibited only a dense, mesh-like structure with reduced pore size.

The structure of a hydrogel dictates its mechanical properties. A low-density matrix with large pores has a low storage modulus. By increasing the BL irradiation time, we can induce denser crosslinking with reduced pore size. RFP photo-crosslinking changed the planar structure to a chemically linked fibrillar one with better mechanical properties.

We examined the changes in the crosslinking of collagen fibrils by BL-activated RFP as a function of irradiation time. The degree of collagen crosslinking increased linearly with irradiation time in both physical and chemical methods. Varying RFP concentrations might affect the crosslinking behavior, but we used a fixed concentration of 0.01% RFP here. Previous studies have revealed that 0.01% is the optimal concentration for RFP as a photosensitizer in collagen solution [15,16].

We confirmed BL-activated RFP-induced collagen crosslinking by collagen hydrogel formation. Collagen is the most abundant protein in the skin (dermis). Therefore, it can form crosslinks in the skin, enhancing its elasticity.

### 3.4. Application of BL-Activated RFP on Porcine Skins

**Figure 5:**
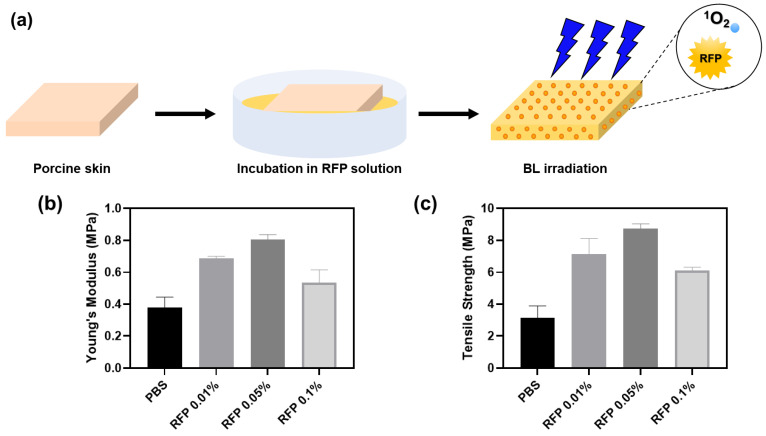
(**a**) Schematics of porcine skin elasticity enhancement by BL-activated RFP. (**b**) Young’s moduli and (**c**) tensile strengths of porcine skins after BL irradiation for 5 min with different RFP concentrations.

We induced BL-activated collagen crosslinking in porcine skin to test for the changes in skin elasticity (Figure 5).

The porcine skin was incubated in an RFP solution for 1 h. The skin color turned slightly yellow upon RFP absorption. We irradiated the skin with BL immediately after incubation and measured its tensile strength and Young’s modulus. In the control group, we incubated the porcine skin in PBS without RFP. Although we confirmed the progress of the reaction by parallelly observing crosslinking in pure collagen solution, the reaction environments in the two cases were different. Therefore, we experimented further to determine the optimal RFP concentration for the porcine skin.

The BL-irradiated skin soaked in RFP solution showed enhanced mechanical properties than the skin incubated in PBS without RFP (Table 2). We observed the highest values of Young’s modulus and tensile strength for skin incubated with 0.05% RFP. The Young’s modulus and tensile strength were approximately 2.1 and 2.8 times higher than those in the control PBS group, respectively. The 0.01% and 0.1% RFP solutions enhanced the mechanical properties but were not as effective as the 0.05% RFP solution. Therefore, 0.05% RFP is optimal for improving skin elasticity. A higher concentration of RFP, say 0.1%, hinders BL penetration, decreasing the effectiveness of the process and resulting in less efficient crosslinking.

The optimal RFP concentrations for skin and collagen solution were 0.05% and 0.01%, respectively. In the collagen hydrogel, all RFP molecules are evenly distributed across the gel and participate in the crosslinking process. However, as all RFP molecules cannot be absorbed in the porcine skin during incubation, we need a higher concentration of RFP to induce efficient crosslinking in the skin matrix. Therefore, we found 0.05% RFP to be the optimal concentration for treating porcine skin.

**Figure 6:**
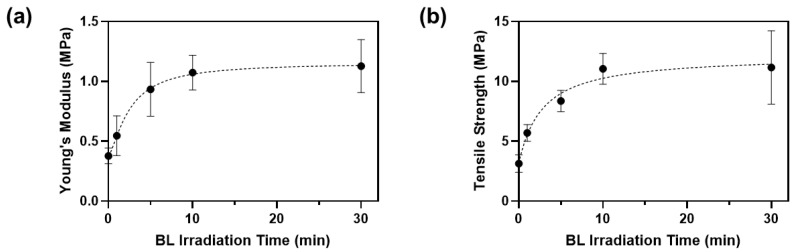
(**a**) Young’s moduli and (**b**) tensile strengths of porcine skins after BL irradiation with 0.05% RFP.

Crosslinking efficiency also depends on the BL irradiation time. We applied BL from 1 to 30 min and measured the changes in mechanical properties of the porcine skin (Figure 6).

The Young’s modulus and tensile strength of porcine skin incubated in PBS without RFP were 0.38 ± 0.05 MPa and 3.14 ± 0.61 MPa, respectively (Table 3). After incubating in the RFP solution and irradiating with BL for 1 min, Young’s modulus and tensile strength increased by 1.4 and 1.8 times, respectively. Overall, the mechanical properties increased with the BL irradiation time until 10 min and plateaued out after that. The rate of increase of the mechanical properties was highest between 5 and 10 min, and the optimal time was approximately 10 min. When both the optimal RFP concentration (0.05%) and BL irradiation time (10 min) were applied, Young’s modulus and tensile strength of porcine skin were 1.07 ± 0.12 MPa and 11.04 ± 1.06 MPa, which were 2.8 and 3.5 times improved compared to those in the control porcine skin, respectively. Thus, the skin elasticity can improve significantly using BL-activated RFP.

The reaction environment during the gelation of collagen solution was simple and well-controlled, but several additional factors need to be considered. Light-activated RFP improves the mechanical properties of the skin by forming bonds between collagen fibrils, but some collagen fibrils also form bonds with other proteins or proteoglycans in tissues [25]. Moreover, since the structure and composition of a skin tissue depends on its origin and varies from person to person, verifying the usability of target tissues is necessary. The corneal crosslinking by RFP has been in use for decades, and various tests evaluating its safety on different target tissues have been conducted. Although the cornea is sensitive to light, photosensitizers are widely used in ophthalmology [13,14]. This indicates that crosslinking using RFP is a safe method, which can be potentially applied to other tissues. Skin is a representative tissue in which we can induce photo-crosslinking. Skin elasticity decreases with age due to reduced or fragmented collagen. RFP can increase collagen crosslinking, repairing the skin and restoring its elasticity.

### 3.5. The Future of BL-Activated RFP

To the best of our knowledge, this is the first study to directly apply RFP to skin tissue. We expect that the collagen photo-crosslinking method will be actively used in skin tissues in the future as it is currently used in the cornea. We increased the safety of the procedure by using BL instead of UV light to activate RFP in this study. With the recent increase in aesthetic devices using LEDs, the light therapy method for skin using RFP is likely to be practically used. Here, we confirmed its potential as a skin-improvement method by testing the increase in the mechanical strength of the skin as a function of collagen crosslinking density. However, additional studies are needed to ensure safer and more efficient use.

Mechanical properties, such as the stiffness of the skin extracellular matrix, play a structural role and affect cell function [26]. Therefore, it is necessary to characterize the mechanical properties of healthy skin. Although the method using light-activated RFP is milder than other photoinitiators, which generate reactive oxygen species, it can induce cytotoxicity when used in actual skin tissue [27]. Therefore, it is necessary to establish an appropriate RFP concentration and protocols based on cell viability and clinical tests.

When collagen is crosslinked by RFP, chemical transformation occurs via the reaction of the carbonyl group, amine group, or specific amino acids. Thus, it is necessary to study the biological changes caused by chemical modifications. Collagen is a component of the extracellular matrix and plays a major role in cell binding. Although collagen crosslinking has been studied using pure collagen, the actual skin tissue environment is different from that of the well-controlled collagen solution, making it necessary to assess the effects of crosslinking on direct cell binding, cell proliferation, and collagen production in the skin. Chemical modification can block the activation site of collagen-degrading enzymes such as collagenase, reducing the enzymatic degradation properties of the crosslinked collagen [28]. Therefore, optimization of skin improvement procedures accounting for changes in cell binding sites and enzymatic degradation sites is required.

This study demonstrated that BL-activated RFP could improve the mechanical strength of pure collagen and porcine skin tissue. Although additional research needs to be conducted before practical application, the combination of RFP and BL irradiation can be potentially used for skin improvement using aesthetic devices.

## 4. Conclusions

We applied RFP-induced photo-crosslinking to the skin to increase its tensile strength and elasticity. BL-activated RFP-induced photo-crosslinking can be used as a skin-enhancement procedure. We modified the corneal crosslinking method used in ophthalmology to increase the collagen crosslinking density of the cornea by using a BL LED instead of UV light to make it safer for the skin. We investigated the changes in dynamic modulus and crosslinked network structures using collagen hydrogels. BL-activated RFP increased the storage and loss modulus of the hydrogels by chemically crosslinking collagen, resulting in dense fibrillar mesh-like structures. The optimum RFP concentration and BL irradiation time were evaluated by applying photo-crosslinking to porcine skin. This study is the first to directly apply RFP-induced photo-crosslinking to skin tissue. We observed a positive effect on the skin elasticity and increased Young’s modulus and tensile strength of the skin. Additional research on diverse tissues to assess the safety of RFP-induced photo-crosslinking is needed before the combination of RFP and BL LED can be utilized as a personal aesthetic skin-enhancement procedure.

## Figures and Tables

**Table 1:** Storage and loss moduli of physically and chemically crosslinked collagen hydrogels.

Group	Storage Modulus (G′, Pa)	Loss Modulus (G″, Pa)
Physical crosslinking	5.28 ± 2.03	2.25 ± 0.60
BL 1 min	8.82 ± 0.72	1.90 ± 0.76
BL 5 min	11.34 ± 1.59	1.72 ± 0.35
BL 10 min	11.96 ± 2.96	2.33 ± 1.88
BL 20 min	17.97 ± 5.22	7.31 ± 1.97
BL 30 min	27.92 ± 3.09	8.53 ± 2.53

**Table 2:** Young’s moduli and tensile strengths of porcine skins by 5-min BL irradiation and different RFP concentrations.

Group	Young’s Modulus (MPa)	Tensile Strength (MPa)
PBS	0.38 ± 0.05	3.14 ± 0.61
RFP 0.01%	0.69 ± 0.01	7.15 ± 0.79
RFP 0.05%	0.80 ± 0.03	8.71 ± 0.27
RFP 0.1%	0.53 ± 0.06	6.11 ± 0.17

**Table 3:** Young’s moduli and tensile strengths of porcine skins by 0.05% RFP and different BL irradiation times.

Group	Young’s Modulus (MPa)	Tensile Strength (MPa)
PBS	0.38 ± 0.05	3.14 ± 0.61
BL 1 min	0.55 ± 0.14	5.69 ± 0.57
BL 5 min	0.93 ± 0.18	8.35 ± 0.73
BL 10 min	1.07 ± 0.12	11.04 ± 1.06
BL 30 min	1.13 ± 0.18	11.15 ± 2.50
